# Supplementary material for: TLR2 and TLR9 Blockade Using Specific Intrabodies Inhibits Inflammation-Mediated Pancreatic Cancer Cell Growth
Source: Antibodies (Basel). 2024 Feb 1;13(1):11. doi: 10.3390/antib13010011 (PMC10885114; doi:10.3390/antib13010011)
Supplement: Supplementary file 1 [file antibodies-13-00011-s001.zip › antibodies-2719161-supplementary.pdf]

Supplementary Data:

Supplementary Methods:

GFP transfection: Transfection of cells was performed using Lipofectamine 3000 reagent (Cat. no. L3000008 Thermo Fisher Scientific) as per manufacturer's protocol. Briefly, BxPC-3 or PANC-1 cells were plated and allowed to adhere overnight. The medium was replaced with OptiMEM, and 0.5 µg pGFP plasmid (Cat. No. 632370, Takara Biocompany) was mixed with 10 µl P3000 in a 250 µl OptiMEM. Thirty microliters of Lipofectamine 3000 were diluted in 250 µl of OptiMEM and incubated at room temperature for 5 min. The plasmid and Lipofectamine 3000 mixture were mixed and incubated at room temperature for 20 min before it was added onto the cells. Cells were incubated at 37^0C for 6 hours and washed thrice. Cells were then used for imaging after 48 hours.

Supplementary Figures:

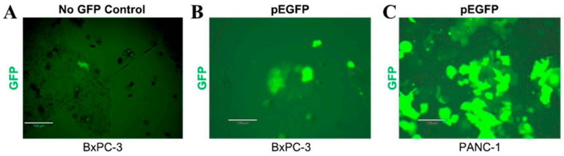

Supplementary Figure S1: PANC-1 shows increased transfection efficiency compared to BxPC-3 cells. (A) Non-transfected BxPC-3 cells. Transfection efficiencies, as shown by transfection of pGFP plasmid, showed increased transfection efficiency in (C) PANC-1 cells compared to (B) BxPC-3 cells. Scale bars = 130 µm.

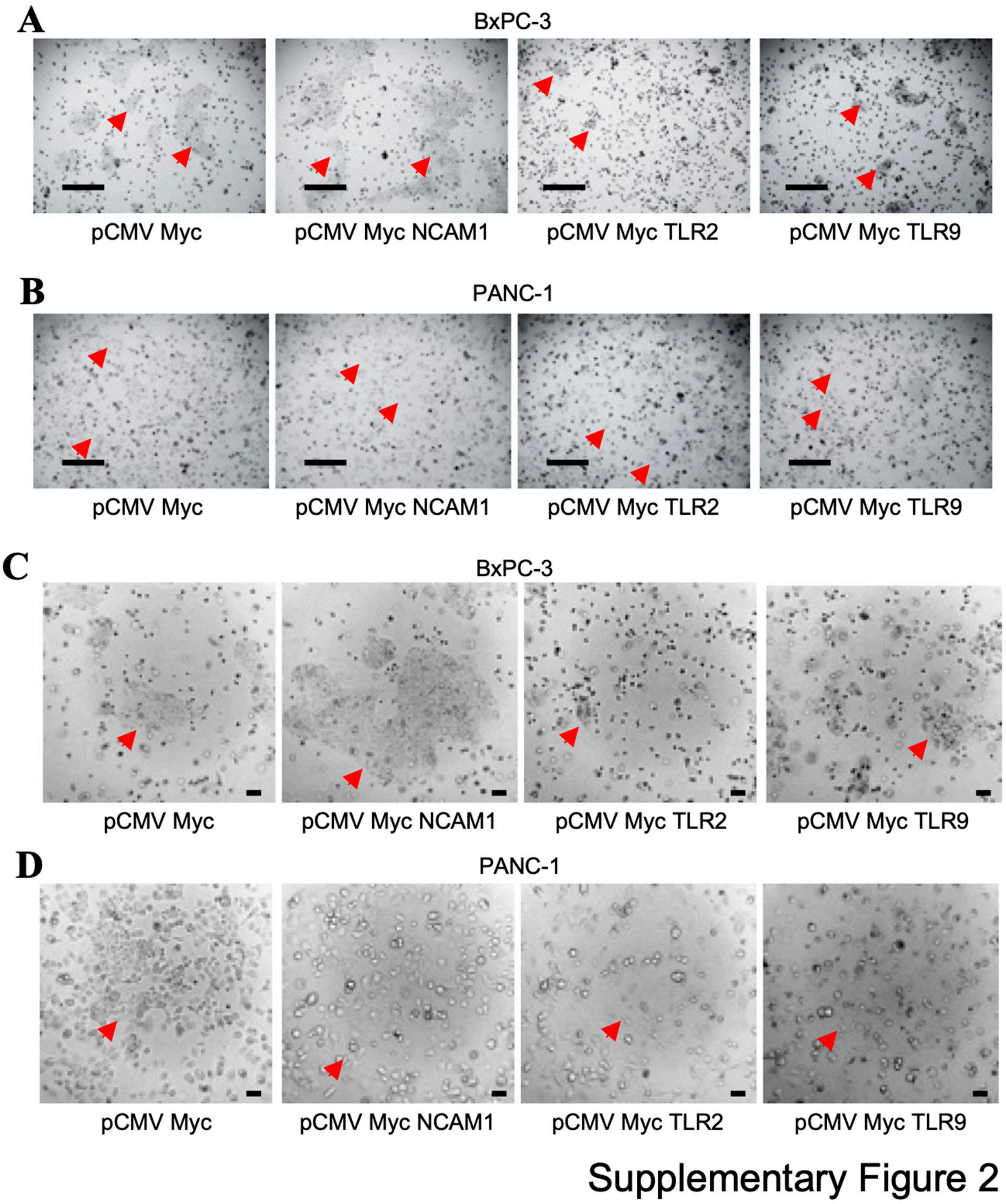

Supplementary Figure S2: TLR2 and TLR9 intrabodies show the enhanced cell death of BxPC-3 (A,C) and PANC-1 cells (B,D)äü. Cells transfected with Myc, Myc

NCAM1, served as control. Images were acquired at 4× magnification (A,B) and 10× magnification (C,D) using phase contrast microscopy. Red arrows indicate the live cells. Scale bars = 320 μm. Phase contrast images showing cell death at 72 h post-transient transfection.
